# Differential expression of matrix metalloproteinases and miRNAs in the metastasis of oral squamous cell carcinoma

**DOI:** 10.1186/s12903-020-1013-0

**Published:** 2020-01-29

**Authors:** Zhen-Hu Ren, Kun Wu, Rong Yang, Zhe-Qi Liu, Wei Cao

**Affiliations:** Department of Oral and Maxillofacial-Head and Neck Oncology, Ninth People’s Hospital, Shanghai Jiao Tong University School of Medicine, No. 639 Zhizaoju Road, Shanghai, 200011 China

**Keywords:** Oral squamous cell carcinoma, Matrix metalloproteinases, Microarray, Metastatic tumour, miRNAs

## Abstract

**Background:**

Our study aimed to reveal the regulatory mechanisms of miRNAs and matrix metalloproteinases (*MMPs*) in oral squamous cell carcinoma (OSCC).

**Methods:**

The mRNA and miRNA expression profiles of six metastatic tumour samples, six nonmetastatic tumour samples, and six normal tissue samples were used for microarray analysis. Moreover, the important genes and miRNAs were validated by published profiles in Oncomine and by qRT-PCR.

**Results:**

*MMP7*, *MMP13*, and *MMP10* were upregulated, and *MMP12* and *MMP9* were downregulated in metastatic tumours compared with nonmetastatic tumours. *MMP7* was regulated by miR-4697-5p and miR-7109-5p. *MMP7* and *MMP13* were upregulated in OSCC samples compared with normal samples in Oncomine. Moreover, qRT-PCR revealed that the expression of miR-7109-5p and miR-34b was decreased in metastatic tumours compared with nonmetastatic tumours.

**Conclusions:**

Our study suggested that miR-7109-5p and miR-34b might play important roles in the metastasis of OSCC by regulating *MMP7* and *MMP13* expression, respectively.

## Background

Oral squamous cell carcinoma (OSCC) is one of the most common malignancies of the head and neck region in the world [1, 2]. OSCC is particularly risky and is usually discovered when the cancer has metastasized to the lymph nodes of the neck since it progresses without producing pain or symptoms that might be readily recognized by the patients in its early stages [3]. Although surgical resection followed by postoperative radiotherapy and/or chemotherapy has made considerable treatment progress, the 5-year overall survival rate of OSCC patients still remains poor due to the common neighbouring tissue invasion and neck lymph node metastasis [4–6]. Moreover, though many research groups have made great efforts to study OSCC pathogenesis, the underlying mechanisms of OSCC tumourigenesis and development have not been fully elucidated. Therefore, further studies focusing on the molecular mechanisms of OSCC are still urgently needed to improve early diagnosis, targeted therapy and prognosis.

Matrix metalloproteinases (MMPs) are a family of highly homologous extracellular zinc- and calcium-dependent endopeptidases with enzymatic activity and are capable of degrading many components from either the extracellular matrix (ECM) or basement membrane [7]. Studies have shown that MMPs are involved in numerous physiopathological processes, such as tissue remodelling, embryonic development, mammary involution, bone reabsorption, and wound healing [8, 9]. MMPs induced by both tumour cells and surrounding stromal cells are related to various processes associated with tumour cell proliferation, angiogenesis, neighbouring invasion and remote metastasis due to their ability to degrade ECM and alter cell migration [10, 11]. Increased levels of one or several MMPs have been found in most human cancers [12]. Overexpressed MMP-2 and MMP-9 are involved in the invasion process of OSCC, and MMP-9 is related to the poor prognosis of OSCC patients without neck node metastasis [13]. Significantly higher MMP-1, MMP-2, MMP-3, MMP-7, MMP-9, MMP-10, MMP-11, and MMP-13 levels were found in tumours compared with normal mucosa, and MMP-9 might be useful for evaluating the malignant potential of head and neck squamous cell carcinoma [14]. It has also been reported that CXCR4 might promote OSCC cell migration and invasion by regulating MMP9 and MMP13 expression to activate the ERK signalling pathway [15]. Overall, increasing evidence has suggested that MMPs play critical roles in OSCC progression.

MicroRNAs (miRNAs) are a class of short noncoding RNAs that regulate the levels of posttranscriptional mRNAs by anchoring to the target sites on mRNA sequences in a complementary base-pairing manner [16, 17]. It has been reported that miR-222 inhibits OSCC cell invasion via the downregulation of MMP1 expression [18]. Moreover, miR-29a may play an inhibiting role in the progression of OSCC by negatively regulating MMP2 expression [19]. These findings provide important clues for potential therapeutic targets and approaches in the future. However, the miRNA regulatory mechanisms of MMP expression in OSCC metastasis remain unclear. Thus, the elucidation of aberrantly expressed MMPs and the related miRNA regulatory mechanisms in OSCC progression is critical.

In the present study, we aimed to reveal the possible regulatory mechanisms related to miRNAs and MMPs involved in the OSCC metastatic process. Samples collected from patients with nonmetastatic or metastatic tumours, as well as normal controls, were used for high-throughput mRNA and miRNA microarray analysis. The selected important markers involved in the metastatic process were also validated by experiments in another cohort of OSCC patients.

## Methods

### Patients and clinical tissue samples

Twenty-seven OSCC patients (14 with nonmetastatic tumours and 13 with metastatic tumours) were enrolled in our retrospective study. The tumours were diagnosed and staged according to the 8th edition of the AJCC/UICC cancer staging manual [20]. All patients underwent ***en bloc*** excision with primary tumour excision combined with neck dissection (bilateral neck dissection was performed if the tumour crossed the midline) in our hospital (Oral and Maxillofacial-Head and Neck Oncology Department, Ninth People’s Hospital, Shanghai Jiao Tong University School of Medicine) between October 2016 and March 2017. The primary tumour site of metastatic OSCC, primary tumour site of nonmetastatic OSCC and paired normal oral mucosa outside the tumour 2 cm away belonged to the same anatomical site as the primary tumour and were collected during surgery (all tissues were cut with a steel knife and immediately snap-frozen in liquid nitrogen). The nonmetastatic patients did not have occult lymph node metastasis discovered postoperatively. The clinical and pathological characteristics of the enrolled patients are listed in Tables 1 and 2. The tumour samples collected from six metastatic OSCC patients (No. 1–6), as well as the tumour and paired adjacent normal tissue samples collected from six nonmetastatic OSCC patients (No. 7–12), were used for the microarray assay. The tumour samples from another eight nonmetastatic OSCC patients (No. 13–20) and seven metastatic OSCC patients (No. 21–27) were obtained for further experimental validation.

**Table 1:** Clinical characteristic of the oral squamous cell carcinoma (OSCC) patients used for microarray assay and qRT-PCR

NO.	Tumor statue	Age range (years)	Site	Clinical stage	TNM classification	Application
1	Nonmetastatic	40–49	Tongue	II	T2N0M0	Microarray assay
2	Nonmetastatic	60–69	Oral floor	III	T3N0M0	Microarray assay
3	Nonmetastatic	40–49	Tongue	II	T2N0M0	Microarray assay
4	Nonmetastatic	60–69	Gingiva	II	T2N0M0	Microarray assay
5	Nonmetastatic	40–49	Tongue	IV	T4N0M0	Microarray assay
6	Nonmetastatic	50–59	Buccal mucosa	III	T3N0M0	Microarray assay
7	Metastatic	50–59	Tongue	III	T2N1M0	Microarray assay
8	Metastatic	60–69	Tongue	IV	T2N2M0	Microarray assay
9	Metastatic	40–49	Tongue	IV	T1N2M0	Microarray assay
10	Metastatic	60–69	Tongue	IV	T2N2M0	Microarray assay
11	Metastatic	50–59	Tongue	IV	T2N2M0	Microarray assay
12	Metastatic	50–59	Buccal mucosa	IV	T1N2M0	Microarray assay
13	Nonmetastatic	50–59	Tongue	II	T2N0M0	qRT-PCR
14	Nonmetastatic	40–49	Tongue	II	T2N0M0	qRT-PCR
15	Nonmetastatic	60–69	Buccal mucosa	II	T2N0M0	qRT-PCR
16	Nonmetastatic	40–49	Gingiva	II	T2N0M0	qRT-PCR
17	Nonmetastatic	30–39	Tongue	II	T2N0M0	qRT-PCR
18	Nonmetastatic	60–69	Gingiva	III	T3N0M0	qRT-PCR
19	Nonmetastatic	70–79	Tongue	III	T3N0M0	qRT-PCR
20	Nonmetastatic	50–59	Tongue	IV	T4N0M0	qRT-PCR
21	Metastatic	50–59	Buccal mucosa	IV	T1N2M0	qRT-PCR
22	Metastatic	70–79	Tongue	IV	T2N2M0	qRT-PCR
23	Metastatic	60–69	Tongue	IV	T2N2M0	qRT-PCR
24	Metastatic	60–69	Tongue	IV	T2N2M0	qRT-PCR
25	Metastatic	50–59	Oral floor	IV	T1N2M0	qRT-PCR
26	Metastatic	50–59	Buccal mucosa	IV	T4N1M0	qRT-PCR
27	Metastatic	40–49	Tongue	IV	T3N1M0	qRT-PCR

*TNM* tumor, *node* metastasis

**Table 2:** Basic information was compared between the two groups

Group	nonmetastatic	metastatic	*P*
Age(y)	55.7	57.9	0.468
Gender			0.157
Man	12	13	
Woman	2	0	
Clinical stage			**0.012**
II	8	0	
III	4	1	
IV	2	12	
Tumor site			0.163
Tongue	8	9	
Buccal	2	3	
Gingiva	3	0	
Oral floor	1	1	

The retrospective study was approved by the Scientific Research Projects Approval Determination of Independent Ethics Committee of Shanghai Ninth People’s Hospital affiliated with Shanghai Jiao Tong University. Informed consent was obtained from all subjects before the samples were collected during surgery.

### Total RNA isolation and microarray processing

Total RNA was extracted from the metastatic tumour, nonmetastatic tumour and normal control tissue samples using TRIzol reagent (Invitrogen, USA) according to the manufacturer’s protocol. Afterwards, the extracted RNA was purified by a Total Tissue RNA Purification Kit (Qiagen Inc., Valencia, CA, USA). The RNA concentrations were measured by a NanoDrop 2000 spectrophotometer (Thermo Scientific, Wilmington, DE, USA). Denaturing gel electrophoresis was used to examine the purity and integrity of the total RNA extracted from the tissue samples.

The qualified total RNA was used to synthetize first-strand cDNA using a cDNA synthesis kit (TIANGEN, China), followed by fluorescent labelling with Agilent’s Low Input Quick Amp WT Labeling kit (Agilent Technologies, USA) according to the manufacturer’s instructions. The labelled cDNA was purified with an RNeasy Mini kit (Qiagen, Germany) and hybridized onto the Agilent Human SurePrint G3 Human GE 8 × 60 k v16 microarray chip (Agilent Technologies, USA) [21] by Shanghai OE Biotech Company (Shanghai, China). Total RNA was also used for the miRNA microarray experiment. The miRNA molecules in total RNA were labelled with the Agilent miRNA labeling reagent and hybridized onto the Agilent Human SurePrint G3 8 × 60 k v16 miRNA microarray chip (Agilent Technologies, USA) according to the manufacturer’s protocol. After hybridization, the samples were scanned by an Agilent Microarray Scanner (Agilent Technologies, USA), and raw data were obtained by using feature extraction software (Agilent Technologies, USA). GeneSpring GX 12.6 software (Agilent Technologies, USA) was used to normalize the obtained raw data.

### Microarray data analysis

The limma (Linear Models for Microarray Analysis) [22] package in R was used to perform differential expression analysis. The thresholds for differentially expressed genes (DEGs) and differentially expressed miRNAs (DE-miRNAs) were set as |log_2_ fold-change (FC)| > 0.5 and *p*-value < 0.05. Moreover, the expression values of screened MMP-related DEGs and DE-miRNAs were hierarchically clustered by the pheatmap package [23] in R based on the encyclopaedia of distances [24] to observe the differences in expression levels intuitively.

The target genes of the differentially regulated miRNAs were predicted by TargetScan (http://www.targetscan.org/vert_71/) [25], miRDB (http://mirdb.org) [26], miRTarBase (http://mirtarbase.mbc.nctu.edu.tw/) [27], and TarBase (http://www.microrna.gr/tarbase) [28]. The miRNA-mRNA regulatory relationships involved with MMPs were selected to construct the MMP-related regulatory network via Cytoscape (http://www.cytoscape.org/) [29]. Furthermore, we used Oncomine (http://www.oncomine.org) to reveal the expression of important genes in the available microarray data comparing OSCCsamples with normal samples.

### Gene ontology (GO) functional and pathway enrichment analysis

The GO functions in terms of the biological process (BP), molecular function (MF), and cellular component (CC) categories for the MMP-related DEGs and target genes of DE-miRNAs were enriched through the Database for Annotation, Visualization and Integrated Discovery (DAVID) [30]. The potential pathways of these MMP-related DEGs and target genes were also revealed by Kyoto Encyclopedia of Genes and Genomes (KEGG) and Orthology-Based Annotation System (KOBAS) [31]. The cutoff criterion was set as *p*-value < 0.05.

### Quantitative real-time RT-PCR (qRT-PCR)

Total RNA was extracted with TRIzol Reagent (Invitrogen, USA), and cDNA was synthesized using the miRcute Plus miRNA First-Strand cDNA Synthesis Kit (TIANGEN, China). All real-time qPCR reactions were performed using an ABI StepOne Real-time PCR system (Life Technologies, USA) and the miRcute Plus miRNA qPCR kit (TIANGEN, China). The threshold cycle calculation of each miRNA was performed with SPSS software version 17.0 (SPSS Inc., Chicago, IL, USA). The samples with undetermined Ct values (Ct > 40) for the control were excluded from analysis. The fold change was assessed using the 2^-ΔΔCt^ method. The relative expression of the miRNAs was normalized using U6 as an endogenous control. Two-sample t test was used to compare the normalized expression levels between the two groups. A *p*-value < 0.05 was considered a statistically significant difference.

## Results

### Identification of MMP-related DEGs and DE-miRNAs

**Fig. 1:**
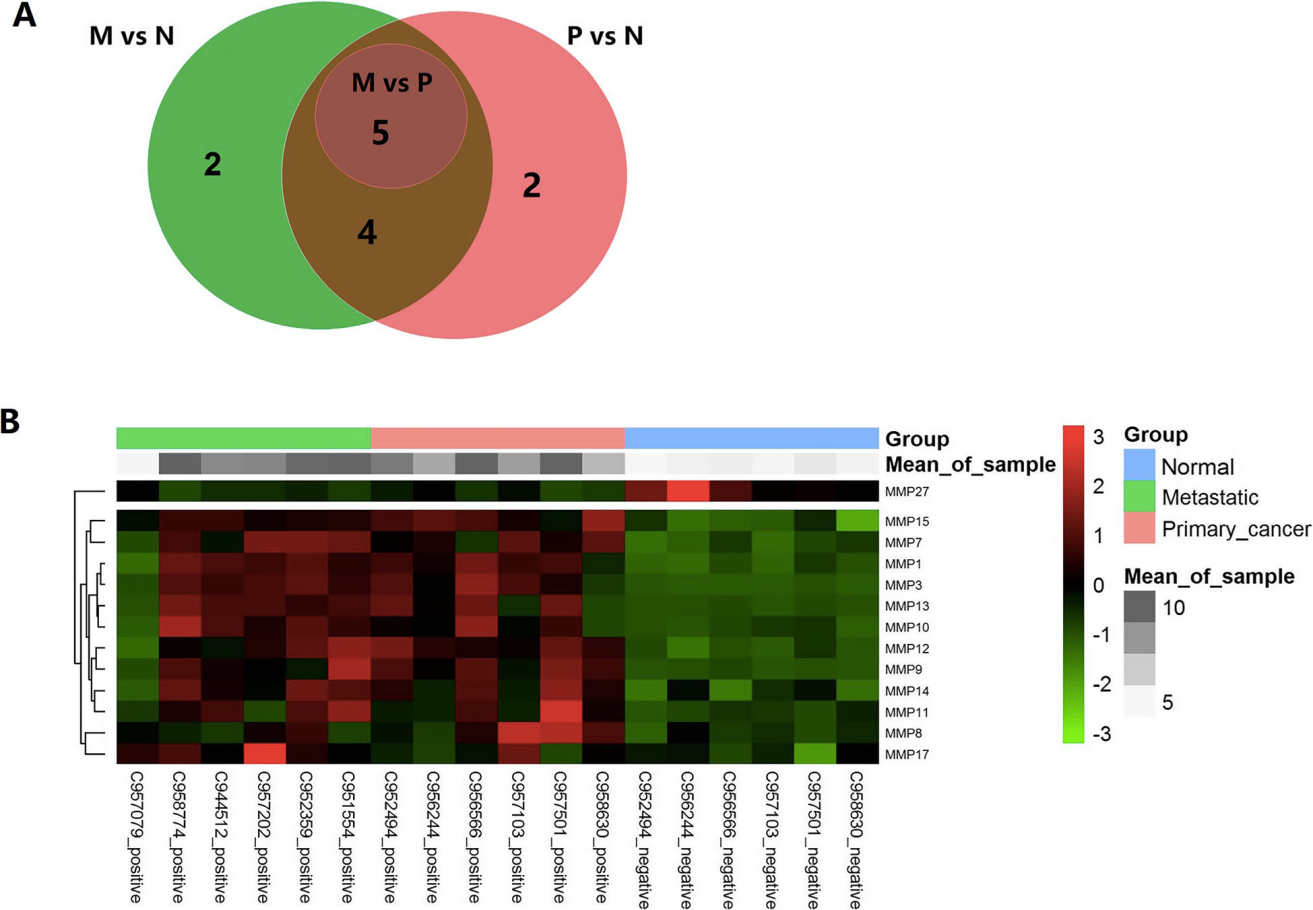
Identification and hierarchical clustering analysis of matrix metalloproteinase (MMP)-related differentially expressed genes (DEGs). **a** MMP-related DEGs in metastatic tumour samples (M vs N) or nonmetastatic oral squamous cell carcinoma (OSCC) samples (P vs N) compared to normal controls and in metastatic tumour samples compared to nonmetastatic cancer samples (M vs P). **b** Hierarchical clustering analysis of 13 MMP-related DEGs in nonmetastatic, metastatic, and normal samples. The black colour represents gene expression that was not altered. The red colour indicates that the gene expression level was increased. The green colour indicates that the gene expression level was decreased

We screened the MMP-related DEGs in metastatic tumour samples (M vs N) or nonmetastatic tumour samples (P vs N) compared to normal controls and in metastatic tumour samples compared to nonmetastatic cancer samples (M vs P) (Fig. 1a). A total of 13 MMP-related DEGs were identified. Ten MMP family members (*MMP12*, *MMP15*, *MMP1*, *MMP9*, *MMP7*, *MMP3*, *MMP14*, *MMP13*, *MMP10*, and *MMP8*) were upregulated and one was downregulated (*MMP27*) in nonmetastatic tumour samples compared with normal controls. Ten MMP family members (*MMP12*, *MMP1*, *MMP9*, *MMP7*, *MMP3*, *MMP14*, *MMP13*, *MMP17*, *MMP10*, and *MMP11*) were upregulated and one was downregulated (*MMP27*) in metastatic tumour samples compared with normal controls. *MMP7*, *MMP13*, and *MMP10* were significantly upregulated, while *MMP12* and *MMP9* were downregulated in metastatic tumour samples compared with nonmetastatic tumour samples. Hierarchical clustering analysis showed that the gene expression profiles were significantly different among nonmetastatic tumour samples, metastatic tumour samples, and normal tissues (Fig. 1) (details in the Additional file 1).

**Fig. 2:**
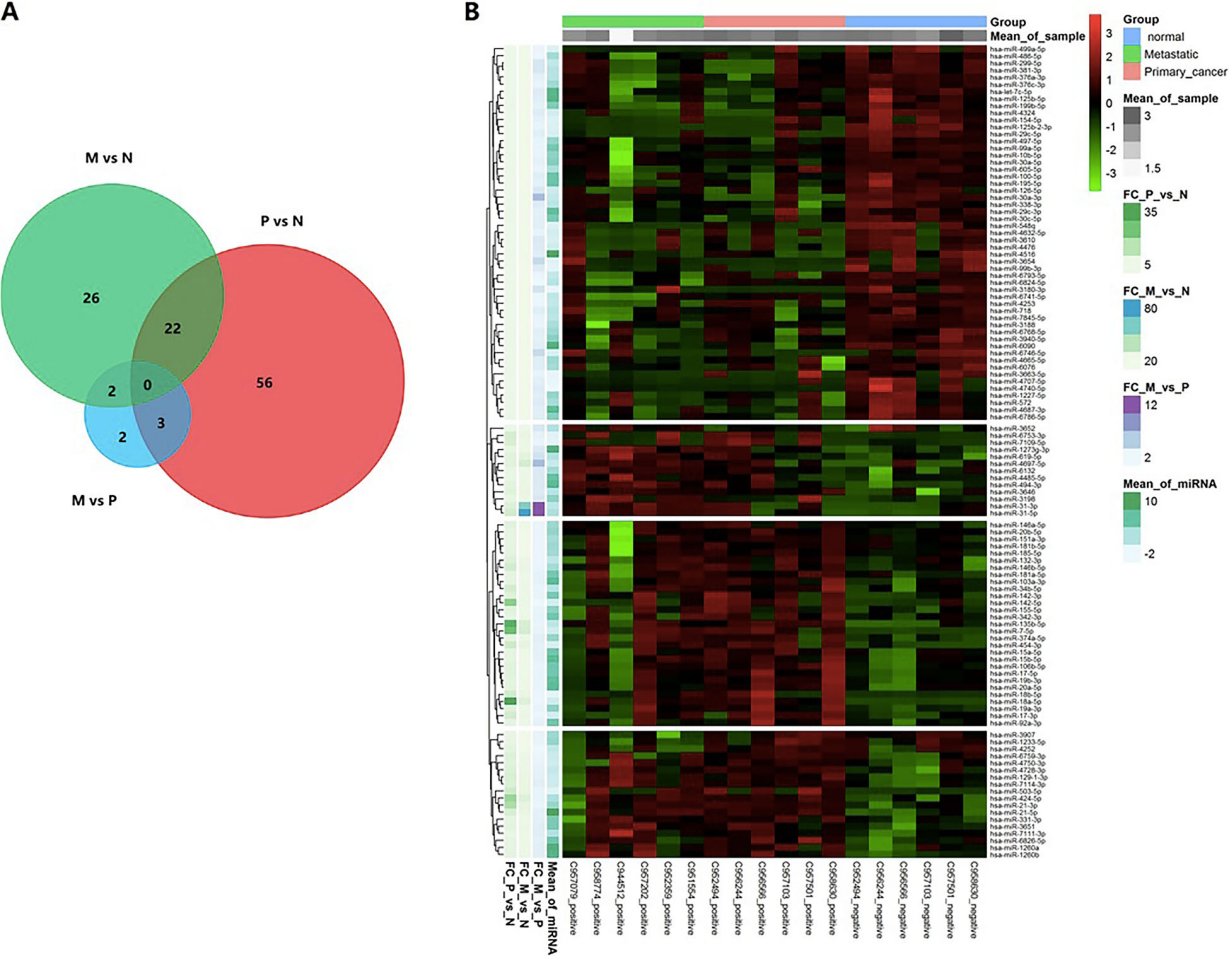
Identification and hierarchical clustering analysis of differentially expressed miRNAs (DE-miRNAs). **a** The DE-miRNAs in metastatic tumour samples (M vs N) or nonmetastatic samples (P vs N) compared to normal controls and in metastatic tumour samples compared to nonmetastatic cancer samples (M vs P). **b** Hierarchical clustering analysis of 111 DE-miRNAs in nonmetastatic, metastatic, and normal samples. The black colour indicates that miRNA expression was not altered. The red colour indicates that the miRNA expression level was increased. The green colour indicates that the miRNA expression level was decreased

According to the differential expression analysis, a total of 111 DE-miRNAs were identified. There were 81 significantly dysregulated miRNAs (35 downregulated and 46 upregulated DE-miRNAs) between nonmetastatic tumour and normal samples (P vs N), 50 significantly dysregulated miRNAs (33 downregulated and 18 upregulated DE-miRNAs) between metastatic tumour and normal samples (M vs N), and 7 significantly dysregulated miRNAs (6 downregulated and 1 upregulated DE-miRNAs) between metastatic and nonmetastatic tumour samples (M vs P) (Fig. 2a). Hierarchical clustering showed obvious miRNA expression differences among nonmetastatic tumour samples, metastatic tumour samples, and normal tissues (Fig. 2b).

### Functional and pathway enrichment analysis

Functional enrichment analysis of the MMP-related DEGs revealed that these genes were notably involved with 38, 4 and 11 GO terms in the BP, CC and MF categories, respectively (Table 3). These functions were mainly involved in the collagen catabolic process (*P* < 0.0001), extracellular matrix disassembly (*P* < 0.0001), extracellular matrix (*P* < 0.0001), and metalloendopeptidase activity (*P* < 0.0001). The three significantly enriched KEGG pathways were bladder cancer, rheumatoid arthritis and pathways in cancer (Table 4).

**Table 3:** The Gene Ontology (GO) functional enrichment analysis in terms of biology process (BP), cellular component (CC), molecular function (MF) for the MMP-related differentially expressed genes (DEGs)

Category	GO_ID	Term	*P* value	Count	Genes
BP	GO:0030574	collagen catabolic process	2.583e-24	11	*MMP7*, *MMP14*, *MMP13*, *MMP1*, *MMP3*, *MMP8*, *MMP9*, *MMP12*, *MMP10*, *MMP11*, *MMP15*
	GO:0032963	collagen metabolic process	2.654e-22	11	*MMP7*, *MMP14*, *MMP13*, *MMP1*, *MMP3*, *MMP8*, *MMP9*, *MMP12*, *MMP10*, *MMP11*, *MMP15*
	GO:0044259	multicellular organismal macromolecule metabolic process	4.247e-22	11	*MMP7*, *MMP14*, *MMP13*, *MMP1*, *MMP3*, *MMP8*, *MMP9*, *MMP12*, *MMP11*, *MMP10*, *MMP15*
	GO:0022617	extracellular matrix disassembly	1.031e-21	11	*MMP7*, *MMP13*, *MMP3*, *MMP12*, *MMP10*, *MMP15*, *MMP14*, *MMP1*, *MMP8*, *MMP9*, *MMP11*
	GO:0044236	multicellular organismal metabolic process	1.445e-21	11	*MMP7*, *MMP13*, *MMP3*, *MMP12*, *MMP10*, *MMP15*, *MMP14*, *MMP1*, *MMP8*, *MMP9*, *MMP11*
	GO:0030198	extracellular matrix organization	1.668e-16	11	*MMP7*, *MMP12*, *MMP10*, *MMP15*, *MMP14*, *MMP8*, *MMP13*, *MMP3*, *MMP1*, *MMP9*, *MMP11*
	GO:0043062	extracellular structure organization	1.720e-16	11	*MMP7*, *MMP12*, *MMP10*, *MMP15*, *MMP14*, *MMP8*, *MMP13*, *MMP3*, *MMP1*, *MMP9*, *MMP11*
	GO:0006508	proteolysis	3.499e-11	12	*MMP7*, *MMP12*, *MMP10*, *MMP14*, *MMP17*, *MMP15*, *MMP13*, *MMP3*, *MMP8*, *MMP9*, *MMP11*, *MMP1*
	GO:0035987	endodermal cell differentiation	3.661e-10	4	*MMP9*, *MMP14*, *MMP15*, *MMP8*
	GO:0001706	endoderm formation	1.064e-09	4	*MMP9*, *MMP14*, *MMP15*, *MMP8*
CC	GO:0031012	extracellular matrix	1.486e-17	12	*MMP7*, *MMP12*, *MMP10*, *MMP15*, *MMP14*, *MMP8*, *MMP13*, *MMP3*, *MMP1*, *MMP17*, *MMP9*, *MMP11*
	GO:0005578	proteinaceous extracellular matrix	4.500e-15	10	*MMP7*, *MMP12*, *MMP10*, *MMP8*, *MMP13*, *MMP3*, *MMP1*, *MMP17*, *MMP9*, *MMP11*
	GO:0005615	extracellular space	7.395e-05	6	*MMP7*, *MMP10*, *MMP13*, *MMP3*, *MMP8*, *MMP9*
	GO:0005576	extracellular region	4.364e-04	10	*MMP10*, *MMP11*, *MMP7*, *MMP17*, *MMP8*, *MMP9*, *MMP3*, *MMP12*, *MMP13*, *MMP1*
MF	GO:0004222	metalloendopeptidase activity	5.214e-25	12	*MMP7*, *MMP14*, *MMP13*, *MMP1*, *MMP3*, *MMP17*, *MMP8*, *MMP9*, *MMP12*, *MMP11*, *MMP10*, *MMP15*
	GO:0008237	metallopeptidase activity	4.690e-22	12	*MMP7*, *MMP13*, *MMP3*, *MMP12*, *MMP10*, *MMP15*, *MMP14*, *MMP1*, *MMP8*, *MMP17*, *MMP9*, *MMP11*
	GO:0004175	endopeptidase activity	7.643e-18	12	*MMP7*, *MMP12*, *MMP10*, *MMP15*, *MMP14*, *MMP8*, *MMP13*, *MMP3*, *MMP1*, *MMP17*, *MMP9*, *MMP11*
	GO:0008233	peptidase activity	1.534e-15	12	*MMP7*, *MMP12*, *MMP10*, *MMP14*, *MMP8*, *MMP17*, *MMP9*, *MMP11*, *MMP15*, *MMP13*, *MMP3*, *MMP1*
	GO:0005509	calcium ion binding	4.088e-12	10	*MMP12*, *MMP10*, *MMP14*, *MMP8*, *MMP17*, *MMP11*, *MMP15*, *MMP13*, *MMP3*, *MMP1*
	GO:0008270	zinc ion binding	4.449e-12	12	*MMP7*, *MMP12*, *MMP10*, *MMP14*, *MMP17*, *MMP15*, *MMP13*, *MMP3*, *MMP8*, *MMP9*, *MMP11*, *MMP1*
	GO:0046914	transition metal ion binding	4.452e-11	12	*MMP7*, *MMP12*, *MMP10*, *MMP14*, *MMP17*, *MMP15*, *MMP13*, *MMP3*, *MMP8*, *MMP9*, *MMP11*, *MMP1*
	GO:0016787	hydrolase activity	2.326e-08	12	*MMP10*, *MMP14*, *MMP15*, *MMP3*, *MMP11*, *MMP7*, *MMP12*, *MMP17*, *MMP13*, *MMP8*, *MMP9*, *MMP1*
	GO:0046872	metal ion binding	7.714e-06	12	*MMP10*, *MMP14*, *MMP15*, *MMP3*, *MMP11*, *MMP7*, *MMP12*, *MMP17*, *MMP13*, *MMP8*, *MMP9*, *MMP1*
	GO:0043169	cation binding	9.747e-06	12	*MMP10*, *MMP11*, *MMP7*, *MMP17*, *MMP8*, *MMP9*, *MMP14*, *MMP15*, *MMP3*, *MMP12*, *MMP13*, *MMP1*
	GO:0003824	catalytic activity	1.878e-04	12	*MMP10*, *MMP11*, *MMP7*, *MMP17*, *MMP8*, *MMP9*, *MMP14*, *MMP15*, *MMP3*, *MMP12*, *MMP13*, *MMP1*

Note: only the top 10 functions related to biology process were shown

**Table 4:** The enriched KEGG (Kyoto Encyclopedia of Genes and Genomes) pathways for MMP-related DEGs

Pathway ID	Description	Count	Genes	*P* Value
hsa05219	Bladder cancer	2	*MMP1*, *MMP9*	3.863e-05
hsa05323	Rheumatoid arthritis	2	*MMP3*, *MMP1*	3.837e-04

**Fig. 3:**
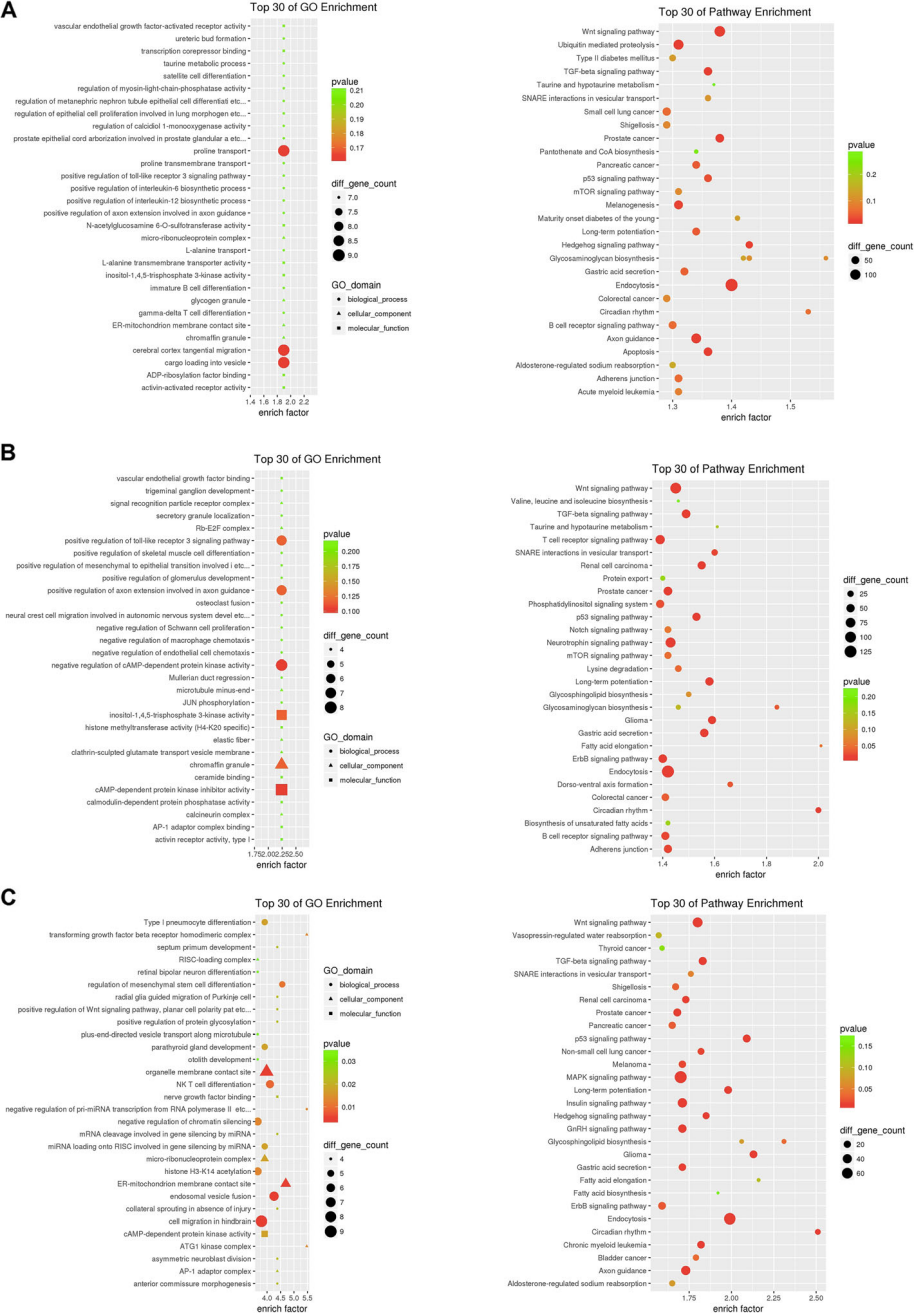
Gene Ontology (GO) functional and pathway enrichment analysis for the target genes of DE-miRNAs. **a** The top 30 GO terms and pathways enriched for the target genes of the DE-miRNAs identified in nonmetastatic tumour samples compared to normal controls. **b** The top 30 GO terms and pathways enriched for the target genes of the DE-miRNAs identified in metastatic tumour samples compared to normal controls. **c** The top 30 GO terms and pathways enriched for the target genes of the DE-miRNAs identified in metastatic tumour samples compared to nonmetastatic tumour samples

The target genes of the three comparison groups of differentially expressed miRNAs were also used to perform functional and pathway enrichment analysis. The target genes of the DE-miRNAs identified in nonmetastatic tumour samples compared to normal controls were mainly enriched in proline transport, cerebral cortex tangential migration, cargo loading into vesicles and endocytosis (Fig. 3a). The target genes of the DE-miRNAs identified in metastatic tumour samples compared to normal controls were mainly enriched in the negative regulation of cAMP-dependent protein kinase activity, cAMP-dependent protein kinase inhibitor activity, and endocytosis (Fig. 3b). The target genes of the DE-miRNAs identified in metastatic tumour samples compared to nonmetastatic tumour samples were mainly enriched in organelle membrane contact site, cell migration in hindbrain, Wnt signalling pathway, TGF-beta signalling pathway, MAPK signalling pathway, and endocytosis (Fig. 3c).

### Construction of the MMP-related regulatory network

**Fig. 4:**
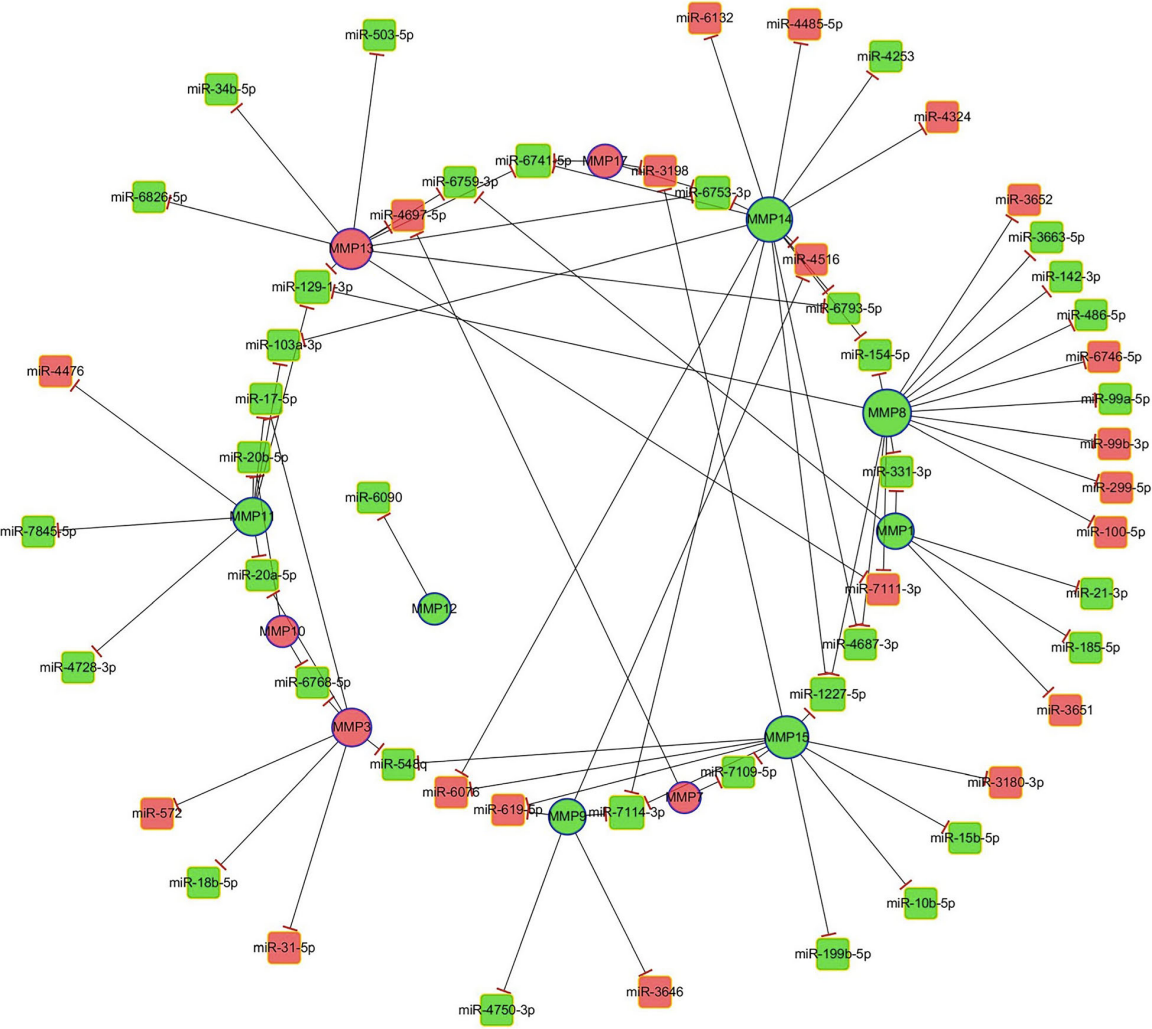
The constructed MMP-related regulatory network. Red nodes represent the upregulated MMP-related DEGs or DE-miRNAs. Green nodes represent the downregulated MMP-related DEGs or DE-miRNAs. Round nodes represent the MMP-related DEGs, and square nodes represent the DE-miRNAs. Lines indicate the regulatory interactions between the MMP-related DEGs and DE-miRNAs

The target genes of the DE-miRNAs were predicted, and the relationships related to MMPs were selected for regulatory network construction. The regulatory network involved 55 DE-miRNAs and 12 MMP-related DEGs (Fig. 4). *MMP7* was regulated by miR-4697-5p and miR-7109-5p. *MMP13* was regulated by 10 DE-miRNAs (miR-4697-5p, miR-6826-5p, miR-6741-5p, miR-6793-5p, miR-34b, miR-6759-3p, miR-503-5p, miR-6753-3p, miR-7111-3p, and miR-129-1-3p). *MMP10* was the target gene of miR-20b-5p and miR-6768-5p. *MMP12* was regulated by miR-6090, and *MMP9* was regulated by 5 DE-miRNAs (miR-619-5p, miR-3646, miR-4750-3p, miR-4516, and miR-7114-3p) (Table 5).

**Table 5:** The MMP-related DEGs and DE-miRNAs in the constructed regulatory network

MMP-related DEGs	Degree	DE-miRNAs
MMP12	1	miR-6090
MMP10	2	miR-20b-5p, miR-6768-5p
MMP7	2	miR-4697-5p, miR-7109-5p
MMP17	3	miR-3198, miR-6741-5p, miR-6753-3p
MMP1	5	miR-21-3p, miR-6759-3p, miR-185-5p, miR-331-3p, miR-3651
MMP9	5	miR-619-5p, miR-3646, miR-4750-3p, miR-4516, miR-7114-3p
MMP3	7	miR-20a-5p, miR-31-5p, miR-17-5, miR-18b-5p, miR-572, miR-6768-5p, miR-548q
MMP11	8	miR-20a-5p, miR-17-5p, miR-103a-3p, miR-4728-3p, miR-20b-5p, miR-7845-5p, miR-129-1-3p, miR-4476
MMP13	10	miR-4697-5p, miR-6826-5p, miR-6741-5p, miR-6793-5p, miR-34b-5p, miR-6759-3p, miR-503-5p, miR-6753-3p, miR-7111-3p, miR-129-1-3p
MMP15	11	miR-619-5p, miR-3198, miR-3180-3p, miR-15b-5p, miR-7109-5p, miR-10b-5p, miR-199b-5p, miR-1227-5p, miR-6076, miR-548q, miR-7114-3p
MMP14	14	miR-6132, miR-4485-5pmiR-103a-3pmiR-6741-5p, miR-6793-5p, miR-4253, miR-154-5p, miR-1227-5p, miR-6753-3p, miR-4324, miR-6076, miR-4516, miR-7114-3p, miR-4687-3p
MMP8	15	miR-3652, miR-3663-5p, miR-142-3p, miR-486-5p, miR-6746-5p, miR-99a-5p, miR-331-3p, miR-154-5p, miR-99b-3p, miR-299-5p, miR-1227-5p, miR-7111-3p, miR-129-1-3p, miR-4687-3p, miR-100-5p

Note: the miRNA names in red color are validated using qRT-PCR

### Validation by data mining and qRT-PCR

**Fig. 5:**
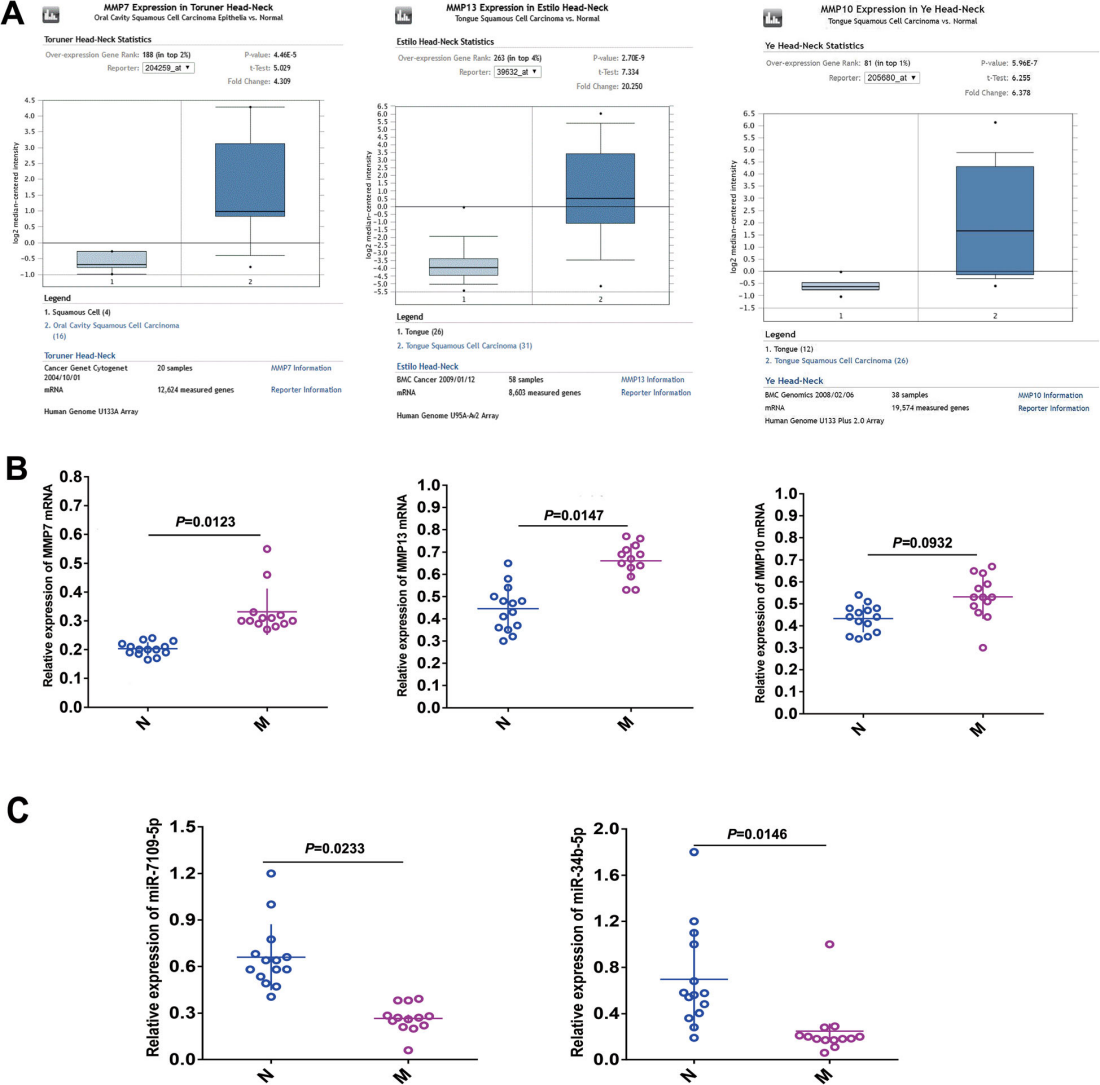
Validation of the important MMP-related DEGs and DE-miRNAs. **a** The expression levels of MMP7 and MMP13 were significantly upregulated in oral cancer compared with normal samples according to the data mining of published profiles in Oncomine. **b** qRT-PCR confirmed that the expression levels of MMP7 and MMP13 were significantly increased in the metastatic tumour samples, while MMP10 was not; **c** qRT-PCR confirmed that the expression levels of miR-7109-5p and miR-34b were significantly downregulated in the metastatic OSCC samples compared with the nonmetastatic tumour samples. The samples were collected from another eight nonmetastatic OSCC patients and seven metastatic OSCC patients

The expression levels of MMP7 (*P* < 0.0001), MMP13 (*P* < 0.0001) and MMP10 (*P* < 0.0001) were also significantly higher in OSCC samples than in normal samples by data mining of published profiles in Oncomine (http://www.oncomine.org) (Fig. 5a). The expression levels of MMP7, MMP13, MMP10, miR-7109-5p and miR-34b in nonmetastatic cancer samples and metastatic cancer samples were measured by qRT-PCR. The expression levels of MMP7 (*P* = 0.0123) and MMP13 (*P* = 0.0147) were significantly increased in the metastatic tumour samples, while that of MMP10 (*P* = 0.0932) was not (Fig. 5b). The expression levels of both miR-7109-5p (*P* = 0.0233) and miR-34b (*P* = 0.0146) were decreased in metastatic tumour samples compared with nonmetastatic tumour samples (Fig. 5c).

## Discussion

In our study, mRNA and miRNA microarrays were used to analyse the expression profiles of MMPs and miRNAs in nonmetastatic tumour samples, metastatic tumour samples, and normal tissues. The results showed that 10 MMP genes were upregulated and *MMP27* was downregulated in nonmetastatic tumour samples compared with normal controls. *MMP12*, *MMP1*, *MMP9*, *MMP7*, *MMP3*, *MMP14*, *MMP17*, *MMP13*, and *MMP10* were the 9 common upregulated genes in metastatic and nonmetastatic tumour samples compared with the normal controls. Moreover, *MMP7*, *MMP13*, and *MMP10* were significantly upregulated in metastatic tumour samples compared with nonmetastatic tumour samples.

Deraz et al. [32] revealed that the overexpression of MMP-10 could promote the invasion and metastasis of head and neck squamous cell carcinoma, and invasion driven by MMP-10 is possibly associated with p38 MAPK inhibition. It has been reported that MMP-13 could promote the invasion, migration, and adhesion abilities of oral cancer cells [33]. The abnormal expression of MMP-7 has been found to be closely related to the biological behaviour of OSCC, and MMP-7 may be induced by COX-2 to contribute to the invasion and metastasis of OSCC [34]. The upregulation of glutamate decarboxylase 1 (GAD1) correlated with cellular invasiveness and migration in OSCC by regulating β-catenin translocation and MMP7 activation [35]. Functional enrichment analysis revealed that *MMP7*, *MMP13*, and *MMP10* were related to the collagen catabolic process, extracellular matrix disassembly, extracellular matrix, and metalloendopeptidase activity. Therefore, the overexpression of *MMP7*, *MMP13*, and *MMP10* might play important roles in controlling tumoural invasiveness and metastasis in OSCC.

The expression levels of MMP7, MMP13 and MMP10 were validated to be significantly upregulated in oral cancer compared with normal samples according to the data mining of published profiles in Oncomine. Among these differentially expressed MMPs, we focused on the roles of MMP7, MMP13 and MMP10. After the target genes of the DE-miRNAs were predicted, functional enrichment analysis showed that the target genes of the DE-miRNAs identified in metastatic tumour samples compared to nonmetastatic tumour samples were mainly enriched in organelle membrane contact sites, cell migration in the hindbrain, and the TGF-beta signalling pathway. TGF-β1-triggered epithelial-mesenchymal transition (EMT) may play important roles in OSCC progression by upregulating MMPs to promote EMT [36]. Thus, the identified DE-miRNAs might be important for the progression of OSCC by regulating the target gene levels to influence the TGF-beta signalling pathway.

In our study, the regulatory relationships related to MMPs were selected to construct a regulatory network. In the regulatory network, *MMP7* was regulated by miR-4697-5p and miR-7109-5p. *MMP13* was regulated by 10 DE-miRNAs (miR-4697-5p, miR-6826-5p, miR-6741-5p, miR-6793-5p, miR-34b, miR-6759-3p, miR-503-5p, miR-6753-3p, miR-7111-3p, and miR-129-1-3p). Moreover, qRT-PCR confirmed that the expression of miR-7109-5p and miR-34b was significantly downregulated in metastatic OSCC samples compared with nonmetastatic tumour samples using samples collected from another cohort of OSCC patients. The abnormal expression of miR-7109-5p has been reported to occur in breast cancer and is associated with cancer development in patients with chronic obstructive pulmonary disease [37, 38]. The reduced expression of miR-34b and miR-129-3p was observed in gastric cancers due to DNA hypermethylation and was associated with poor clinicopathological features [39]. It was also suggested that the reduced expression of miR-34b*/c may be particularly important for the progression to the most advanced stages of human epithelial ovarian cancer [40]. Low expression levels of miR-34b and miR-34c were associated with distant metastasis formation in lung cancer [41]. To our knowledge, few studies have reported the regulatory mechanisms of miR-7109-5p and miR-34b in OSCC. According to the results of our study, miR-7109-5p and miR-34b seem to have tumour suppressor functions in OSCC. The aggressive form of OSCC shows the downregulation of these two miRNAs and subsequent upregulation of MMP 7 and MMP13, which are normally inhibited by miR-7109-5p and miR-34b. The feasibility of miR-7109-5p and miR-34b as promising prognostic and diagnostic indicators or potential cancer therapeutic targets will be evaluated in further studies. In the future, we will focus on the interaction of miRNAs and MMPs and the mechanism of OSCC progression mediated by the miRNA axis to provide valuable strategies for the diagnosis, treatment and prognosis of OSCC.

Several limitations of this study should be addressed. The most important limitation in this regard is the limited number of study samples and inability to control for potential confounders. Another limitation is that the patients included in this study had varying T and N stages, and the two groups of cases were not selected in pairs. The homogeneity of the two groups of cases was different. The metastatic group appears to be more homogeneous than the nonmetastatic group. The results of this study are at the transcriptional level, and further experiments at the protein level are needed to verify the results of this experiment.

## Conclusion

In conclusion, our study performed gene and miRNA microarray analysis to reveal the underlying regulatory mechanisms of miRNAs and MMPs involved in the OSCC metastatic process. The MMP-related DEGs and DE-miRNAs were identified among the nonmetastatic tumour samples, metastatic tumour samples, and normal tissues. *MMP7*, *MMP13*, and *MMP10* were upregulated in metastatic tumours compared with nonmetastatic tumours. The reduced expression of miR-7109-5p and miR-34b might play important roles in the metastasis of OSCC by upregulating *MMP7* and *MMP13*, respectively.

## Supplementary information

**Additional file 1:** The raw data of Microarray analysis.

## Data Availability

The datasets used during the current study are available from supplementary file.

## Abbreviations

AJCC/ UICC: American Joint Committee on Cancer/Union for International Cancer Control
DEGs: Differentially expressed genes
DE-miRNAs: Differentially expressed miRNAs
ECM: Extracellular matrix
EMT: Epithelial-mesenchymal transition
miRNAs: MicroRNAs
MMPs: Matrix metalloproteinases
OSCC: Oral squamous cell carcinoma
